# Tumor-induced myeloid-derived suppressor cells promote tumor progression through oxidative metabolism in human colorectal cancer

**DOI:** 10.1186/s12967-015-0410-7

**Published:** 2015-02-01

**Authors:** Li-Ying OuYang, Xiao-Jun Wu, Shu-Biao Ye, Rong-xin Zhang, Ze-Lei Li, Wei Liao, Zhi-Zhong Pan, Li-Min Zheng, Xiao-Shi Zhang, Zhong Wang, Qing Li, Gang Ma, Jiang Li

**Affiliations:** State Key Laboratory of Oncology in South China, 651 Dongfeng East Road, Guangzhou, 510060 China; Collaborative Innovation Center of Cancer Medicine, 651 Dongfeng East Road, Guangzhou, 510060 China; Department of Biotherapy, 651 Dongfeng East Road, Guangzhou, 510060 China; Intensive Care Unit Department, 651 Dongfeng East Road, Guangzhou, 510060 China; Department of Colorectal Surgery, 651 Dongfeng East Road, Guangzhou, 510060 China; School of Pharmaceutical Sciences, 651 Dongfeng East Road, Guangzhou, 510060 China; Center for Cellular and Structural Biology, Sun Yat-Sen University Cancer Center, 651 Dongfeng East Road, Guangzhou, 510060 China

**Keywords:** Myeloid-derived suppressor cells, Colorectal carcinoma, Radical resection

## Abstract

**Background:**

Expansions of myeloid-derived suppressor cells (MDSCs) have been identified in human solid tumors, including colorectal cancer (CRC). However, the nature of these tumor-associated MDSCs and their interactions with tumor cells in CRC are still poorly understood.

**Methods:**

The percentages and phenotype of MDSCs in peripheral blood and tumorous and paraneoplastic tissues from CRC patients, as well as the clinical relevance of these MDSCs, were assessed. Age-matched healthy donors were included as controls. The interaction between MDSCs and T cells or tumor cells was investigated in a coculture system *in vitro*, and the molecular mechanism of the effect of MDSCs on T cells or tumor cells was evaluated.

**Results:**

We discovered that CRC patients had elevated levels of CD33^+^CD11b^+^HLA-DR^−^ MDSCs in primary tumor tissues and in peripheral blood, and the elevated circulating MDSCs were correlated with advanced TNM stages and lymph node metastases. Radical resection significantly decreases the proportions of circulating MDSCs and CD4^+^CD25^high^FOXP3^+^ regulatory T cells. *In vitro*, CRC cells mediate the promotion of MDSC induction. Moreover, these tumor-induced MDSCs could suppress T cell proliferation and promote CRC cell growth via cell-to-cell contact. Such effects could be abolished by the inhibition of oxidative metabolism, including the production of nitric oxide (NO), and reactive oxygen species (ROS).

**Conclusions:**

Our results reveal the functional interdependence between MDSCs, T cells and cancer cells in CRC pathogenesis. Understanding the impact of MDSCs on T cells and tumor cells will be helpful to establish an immunotherapeutic strategy in CRC patients.

**Electronic supplementary material:**

The online version of this article (doi:10.1186/s12967-015-0410-7) contains supplementary material, which is available to authorized users.

## Background

Colorectal cancer (CRC) is the fourth most commonly diagnosed cancer worldwide and is a major cause of cancer-related deaths [1,2]. Numerous studies have demonstrated a link between chronic inflammation and different cancers, including CRC. The intestinal mucosa is normally maintained in a state of controlled inflammation in which there is equilibrium between protective immunity and tolerance to self-antigens and commensal bacteria [3,4]. An imbalance in the immune tumor microenvironment is one risk factor for CRC, and the presence of tumor-infiltrating lymphocytes (TILs) could predict the clinical outcomes of patients with CRC [5-7]. Accumulating evidence indicates that subsets of immune cells, including cytotoxic T cells (CTLs), natural killer (NK) cells, and regulatory T cells (Tregs), play important roles in the progression of CRC and are associated with tumor invasiveness, clinical stage, and patient survival [8-10].

Recent studies suggested that myeloid-derived suppressor cells (MDSCs) play important roles in cancer patients. MDSCs are an immature population of myeloid cells that are present in the majority of cancer patients [11]. MDSCs usually include two major subpopulations: monocytic MDSCs (M-MDSCs) and granulocytic MDSCs (G-MDSCs) [12,13]. In humans, MDSCs constitute a heterogeneous cell population that is not well characterized, partially because no unified markers are currently available for these cells. However, these cells typically express the common myeloid markers CD33 and CD11b but lack markers of mature myeloid cells, such as HLA-DR. Among human MDSCs, the monocytic subset comprises CD14^+^ cells, and the granulocytic subset comprises CD14^−^CD15^+^ cells [14].

A number of studies have shown that MDSCs are correlated with the development of malignancies. Increased numbers of MDSCs have been observed in various solid and hematologic malignancies, and elevated MDSCs are associated with cancer progression and tumor-induced immune dysfunction [13,15-19]. MDSCs could inhibit anti-tumor immunity by suppressing T cell and NK cell functions [20,21], likely by increasing the production of arginine, reactive oxygen species (ROS), and nitric oxide (NO) and by inducing Treg cells and TGF-β secretion to mediate T cell suppression [19,22-24]. In addition to suppressing anti-tumor immunity, the direct interaction between MDSCs and tumor cells has been explored in recent years [25]. However, the specific signals triggering the accumulation of MDSCs in cancer patients remain unidentified, and the mechanism of the tumor-induced MDSC function in CRC is not yet well defined.

In this study, we examined the correlation between MDSCs and tumor progression in CRC patients and during routine treatment. We also studied how MDSCs interact with T cells and CRC cells *in vitro*.

## Materials and methods

### Samples and cell lines

Peripheral blood was collected from 32 age-matched healthy donors and 42 patients with CRC at the time of first diagnosis at Sun Yat-Sen University Cancer Center, Guangzhou, China, from March 2013 to June 2013; these patients did not receive any pre-operative chemoradiotherapy. The clinical details of the patients are provided in Additional file 1: Table S1. Peripheral blood was collected from 26 of the 42 patients who did not show any signs of infection, inflammation, or other complications before or after tumor curative resection one week after the operation. Fresh tumor and paraneoplastic tissues were obtained from 19 of the patients. All the tumor tissue samples were removed by surgical resection; three were collected by palliative resection, and 16 were collected by curative resection. All of the patients and healthy donors provided written informed consent before being subjected to blood sampling and/or tumor harvesting. This study was approved by the Research Ethics Committee of the Sun Yat-Sen University Cancer Center.

Peripheral blood mononuclear cells (PBMCs) were isolated via Ficoll-Hypaque gradient centrifugation to measure the proportion and phenotype of MDSCs. Fresh tumor and paracancerous tissues were cut into pieces; one piece was frozen at −80°C for subsequent qRT-PCR or immunochemical staining, and the other piece was enzymatically digested with type IV collagenase for 2 hours at 37°C in a water bath oscillator. Bulk cells from tissues were cultured in complete RPMI 1640 medium (Gibco, Life Technologies, USA) supplemented with heat-treated 10% fetal bovine serum (FBS, exCell Biology, South America) without IL-2 overnight to obtain a sufficient number of cells for FACS analysis.

The human SW480 and SW620 colorectal cancer cell lines were maintained in our laboratory and cultured in RPMI 1640 supplemented with 10% FBS and gentamicin sulfate (Guangdong Succhi Shiqi Pharmaceutical, China).

### Antibodies and fluorescence-activated cell sorting (FACS) analysis

Human monoclonal antibodies against HLA-DR, CD4, CD33, CD11b, CD45, CD8, CD25, CD3, CD16, FOXP3, CD14, CD15, CD39, CD73, CD117, CD66b, CXCR4, CD34, arginase1 (ARG1), iNOS and PD-L1 conjugated with different fluorescent dyes were purchased from BD Pharmingen (San Jose, CA, USA) or eBioscience (San Diego, CA, USA). The oxidation-sensitive dye CM-H2DCFDA was purchased from Invitrogen (Grand Island, NY).

The expression of markers on the MDSCs and tumor cells was investigated using FACS analysis after surface staining or intracellular staining with human-specific Abs conjugated with different fluorescent dyes. The MDSC phenotype analysis involved gating within the HLA-DR^−^ cell population that expressed both the CD33 and CD11b antigens. Intracellular staining to detect Foxp3 was performed on T cells from PBMCs, tumorous tissues, and paracancerous tissues. After washing, the cells were stained with anti-CD4, fixed, permeabilized with Perm/Fix solution (eBioscience), and stained intracellularly with anti-Foxp3. The cells showing positive staining were detected using the Cytomics FC 500 MPL flow cytometry system (Beckman Coulter) and analyzed with CXP software (Beckman Coulter, Inc. Fullerton, CA, USA).

### Induction of MDSCs and functional analysis of tumor-induced MDSCs *in vitro*

CD33^+^ cells were separated from healthy PBMCs using human CD33 MicroBeads (Miltenyi Biotec, Bergisch Gladbach, Germany) according to the manufacturer’s instructions. Isolated CD33^+^ cells were co-cultured with SW480 or SW620 cells in 24-well plates in a Transwell System at a ratio of 1:5 for 48 h. CD33^+^ cells cultured in medium alone were included as a control. A panel of harvested cells was used to analyze the MDSC markers using FACS, and the remaining cells were subjected to the proliferation assay. In brief, PBMCs from healthy donors were labeled with CFSE (5 μM) and added in different ratios to the induced MDSCs in OKT3-coated 96-well plates. The cells were cultured for 3 days. The experiments included a panel of samples treated with N^G^-Monomethyl-L-arginine (1 mM/mL), L-NG-Monomethylarginine (L-NMMA, 100 μm/mL), or N-acetylcysteine (NAC 1 mM/mL, Sigma, Saint Louis, MO, USA), which lead to arginine starvation and inhibition of the generation of NO and ROS, or with human TGF-β neutralizing antibody (10 μg/mL; R&D Systems, Minneapolis, MN, USA) [26,27]. These samples were analyzed using FACS.

The proliferation of SW480 cells with co-cultured tumor-induced MDSCs was assessed by counting the cells from day 1 to day 5 directly, in a Transwell System, or alone in medium. This experiment included a panel of samples treated with supplementary L-arginine, L-NMMA, and NAC.

### Semi-quantitative RT-PCR (qRT-PCR)

Total RNA from cells or from frozen tumorous and paracancerous tissues was extracted using the TRIzol reagent (Invitrogen, USA) according to the manufacturer’s instructions. RT-PCR was performed with the RevertAid First-Strand cDNA Synthesis kit (Thermo Scientific, Lithuania) using Premix Taq™ (TaKaRa Taq™ Version 2.0 plus dye). The primer sequences are given in Additional file 1: Table S2. The obtained PCR products were examined via agarose gel electrophoresis. All of the tests were repeated at least 5 times, and GAPDH mRNA was used as a control.

### Statistical analysis

All of the *in vitro* experiments were performed in triplicate and were repeated at least three times. Representative experiments are shown in the figures. The statistical analysis was performed with SPSS 13.0 software (SPSS, Chicago, IL, USA). Numerical data are shown as means ± standard error of the mean (SEM). Comparisons between two groups were tested using Student’s *t* test, and the association of the density of MDSCs with the clinical pathologic features was examined using a Pearson *χ*^2^ test and Fisher's exact test. In all of the analyses, the threshold for significance was *P* < 0.05.

## Results and discussion

### Results

#### MDSCs are expanded in CRC patients

**Figure 1:**
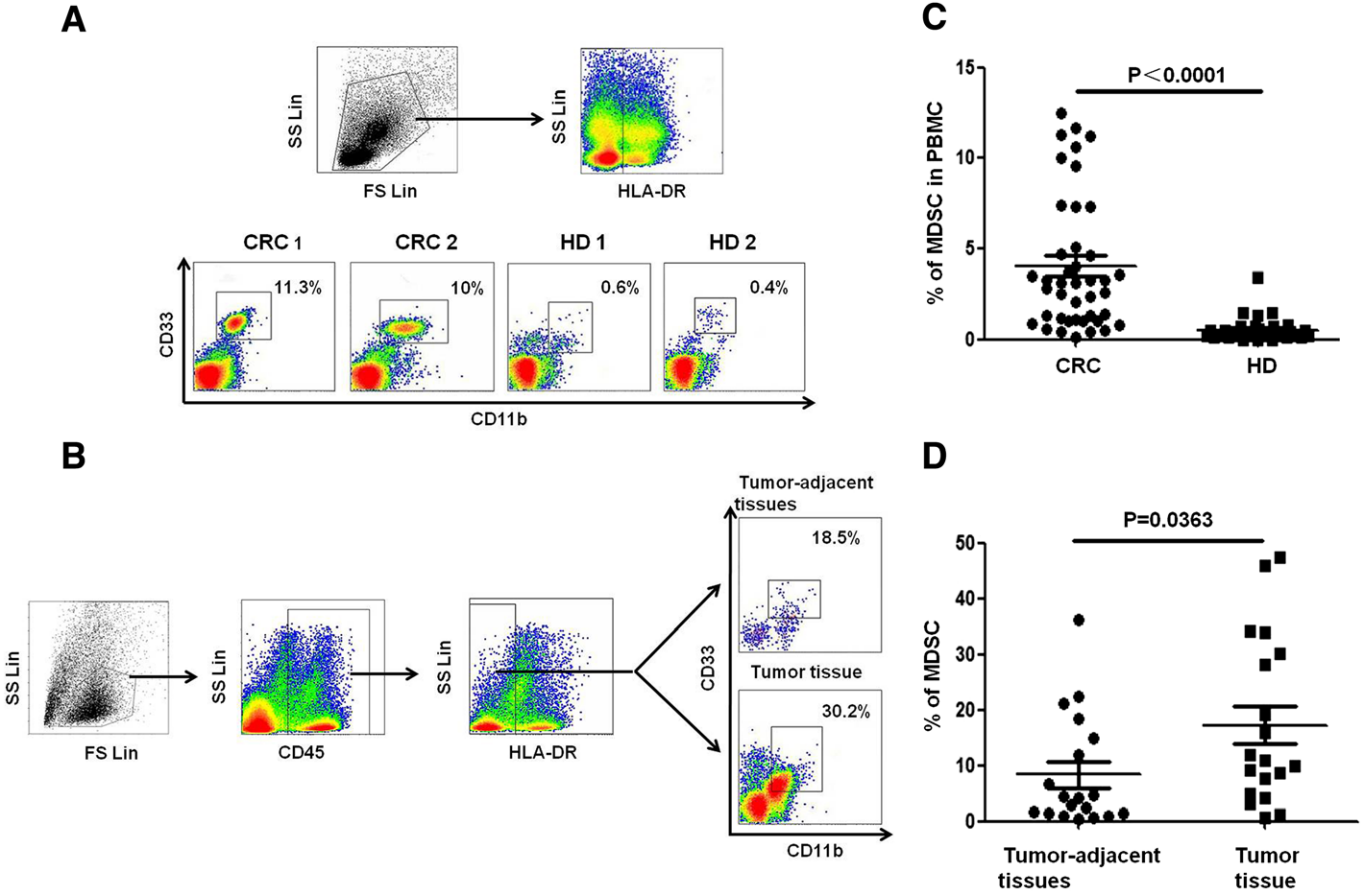
**Distribution of MDSCs in patients with CRC. (A)** Gating strategy for the assessment of the MDSC population using flow cytometry. HLA-DR^−^ cells were gated from live PBMCs, and CD33^+^CD11b^+^ cells were further gated as MDSCs. Representative density plots from two CRC patients and healthy donors are shown. **(B)** The dot plots represent CD33^+^CD11b^+^ MDSC gating from the HLA-DR^−^CD45^+^ cell fraction from the tumorous and paraneoplastic tissues of one CRC patient. **(C)** Statistical analysis of the percentage of MDSCs among the PBMCs from CRC patients (n = 42) and healthy donors (n = 32). **(D)** The proportion of MDSCs was significantly increased in tumorous tissues compared with paraneoplastic tissues and PBMCs from CRC patients (n = 19). Error bars represent SEM.

Base on previous reports [28-30], in the current study, we investigated the proportion and distribution of MDSCs immunophenotyped as CD33^+^CD11b^+^ HLA-DR^−^ cells in CRC patients (n = 42) and compared this with those of healthy donors (n = 32). The proportion of MDSCs in peripheral blood from pre-treated CRC patients was significantly increased compared with the proportion from healthy donors (*P* < 0.05). To explore the distribution of MDSCs in CRC patients, we further assessed the proportion of MDSCs in the tumorous tissues and tumor-adjacent tissues from 19 paired CRC patients. The proportion of MDSCs was higher in the tumorous tissues compared with tumor-adjacent tissues (*P* < 0.05), as shown in Figure 1.

**Figure 2:**
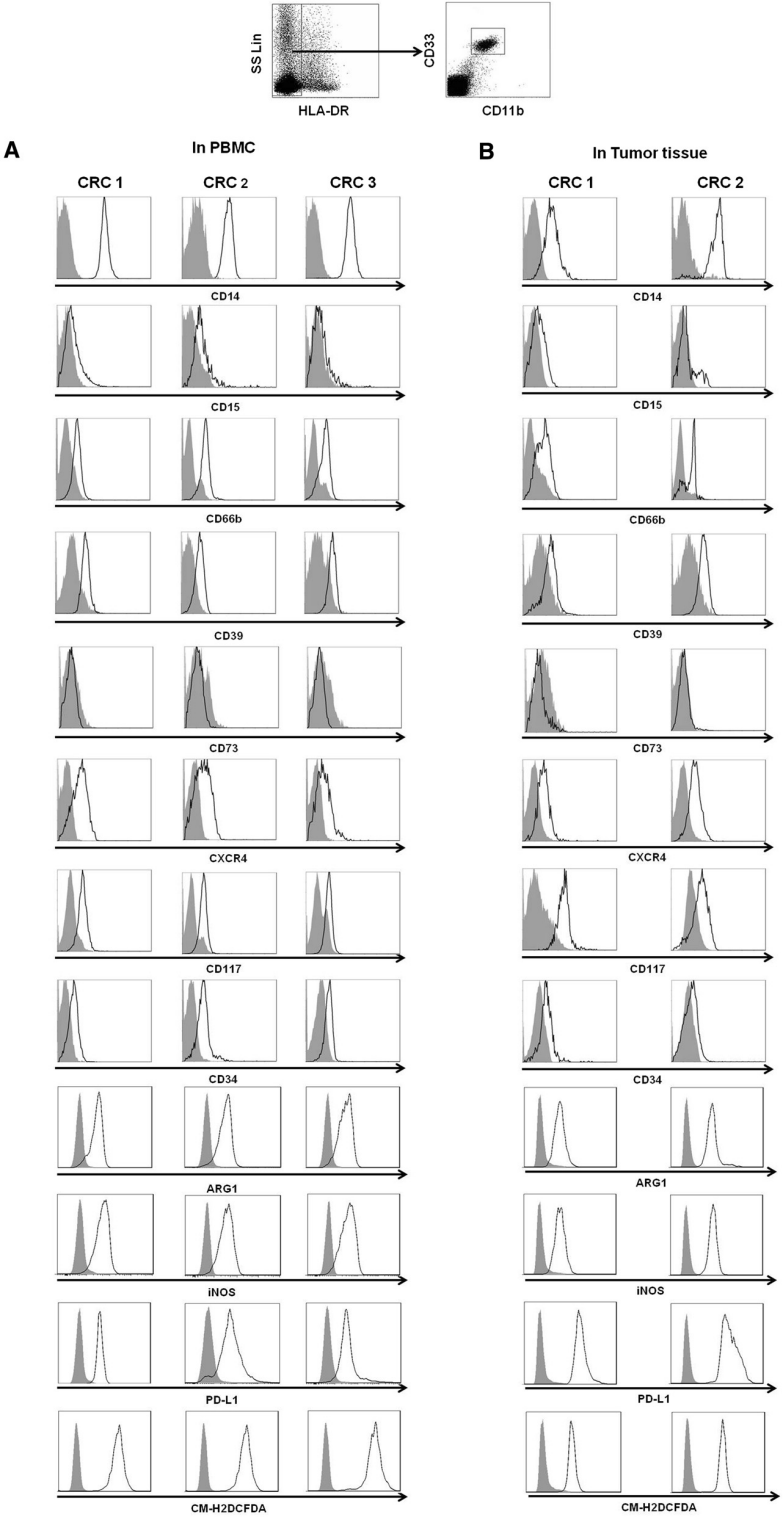
**Phenotypes of HLA-DR** ^**−**^ **CD33** ^**+**^ **CD11b** ^**+**^ **MDSCs in CRC patients.** HLA-DR^−^CD33^+^CD11b^+^ cells were gated as MDSCs from either PBMCs **(A)** or tumor tissues **(B)** from CRC patients. The phenotypes of the MDSCs were analyzed with flow cytometry using multiple anti-human mAbs against CD14, CD15, CD66b, CD39, CD73, CXCR4, CD117, CD34, ARG1, INOS, PD-L1 and DCFDA as indicated, and the grey curve represents autofluorescence as a negative control. Representative histograms are shown.

We examined the expression levels of phenotypic markers in this CD33^+^CD11b^+^HLA-DR^−^ MDSC population from CRC patients, including the levels of CD14, CD15, CD66b, CD117, CD73, CD39 CD34, CXCR4, iNOS, ARG-1, PD-L1 and ROS (Figure 2). The MDSC population expressed high levels of the monocytic marker CD14, the chemokine receptor CXCR4, and the ectonucleoside triphosphate diphosphohydrolase 1 (ENTPD1) CD39, was well as immune mediators including ARG-1, iNOS, ROS and the suppressive immune checkpoint molecule PD-L1 but did not express the ecto-5'-nucleotidase CD73; these molecules are involved in the degradation of immunostimulatory ATP into immunosuppressive adenosine. The MDSC population expressed moderate levels of the granulocyte-monocyte progenitor cell markers CD117, CD34, and CD66b and expressed a weak level of the granulocytic marker CD15. The MDSCs from the peripheral blood and tumorous tissue of CRC patients exhibited similar phenotypic characteristics.

#### Elevated circulating MDSCs are associated with advanced disease stage in CRC patients

To investigate the clinical relevance of MDSCs in CRC patients, we determined the correlations between the proportion of MDSCs among PBMCs and the clinical characteristics of the CRC patients, including age, gender, TNM stage, tumor size, lymph node metastasis, histological grade, venous invasion and serum CEA level (Table 1). An increased proportion of MDSCs among PBMCs was associated with an advanced TNM stage and with lymph node metastases (*P* = 0.020 and 0.008, respectively), whereas no correlation between the proportion of MDSCs in PBMCs and the other clinical characteristics, including tumor size, histological, and serum CEA level, of patients were found.

**Table 1:** **Correlations between the proportion of circulating MDSCs and the clinical characteristics of CRC patients**

**Characteristics**	**No. of patients**	**Mean of circulating MDSCs**		***P*** **value**
		**Low (≤4.0%)** ^**a**^	**High (>4.0%)** ^**a**^	
**Age (years)**				
≤60	27	16	11	
>60	15	11	4	0.506
**Gender**				
Male	20	12	8	
Female	22	15	7	0.749
**TNM stage**				
I-II	24	19	5	
III-IV	18	8	10	0.020*
**T stage**				
**T2**	3	1	2	
**T3**	28	20	8	
**T4**	11	6	5	0.312 (Fisher’s)
**Tumor size (cm)**				
≤5	25	15	10	
>5	17	12	5	0.531
**Lymph node metastasis**				
No	26	21	5	
Yes	16	6	10	0.008**
**Histological grade**				
Well/moderate	24	18	6	
Poor/undifferentiated	18	9	9	0.116
**Venous invasion**				
No	38	26	12	
Yes	4	1	3	0.122
**Serum CEA (ng/mL)**				
≤5	22	16	6	
>5	20	11	9	0.336

Note: ^a^4.0% indicates the mean percentage of circulating MDSC; *indicates *P* < 0.05; **indicates *P* < 0.01; determined by Pearson *χ* ^2^ test and Fisher’s exact test.

#### Tumor resection reduces the circulating MDSCs and Tregs in CRC patients

**Figure 3:**
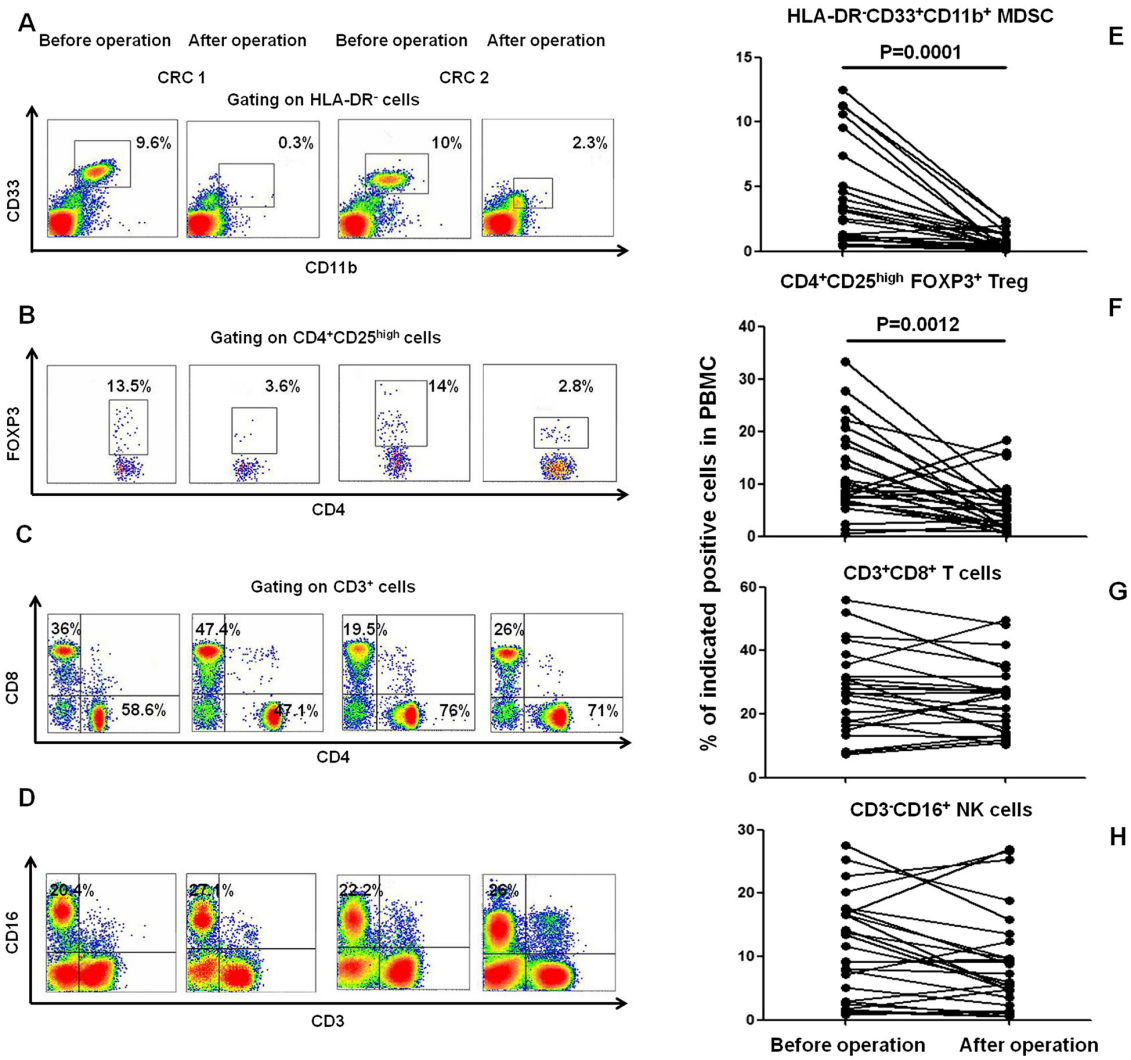
**Decreased proportions of circulating HLA-DR** ^**−**^ **CD33** ^**+**^ **CD11b** ^**+**^ **MDSCs and CD4** ^**+**^ **CD25** ^**high**^ **FOXP3** ^**+**^ **Treg cells in CRC patients after tumor resection.** PBMCs were isolated from 26 CRC patients before and after surgery. **(A-D)** Representative density plots of circulating HLA-DR^−^CD33^+^CD11b^+^ MDSCs, CD4^+^CD25^high^FOXP3^+^ Tregs, CD3^+^CD4^+^ T cells, CD3^+^CD8^+^ T cells, and CD3^−^CD16^+^ NK cells from one CRC patient before and after surgery are shown. **(E-H)** The graph shows that the proportions of circulating MDSCs and Tregs were significantly decreased after surgery (*P* < 0.05), whereas the proportions of CD3^+^CD4^+^ T cells, CD3^+^CD8^+^T cells, and CD3^−^CD16^+^ NK cells were not significantly different after the operation (*P* > 0.05).

Surgery remains the main treatment option for CRC patients. However, the effect of surgery on the host immune status has not been well studied. To illustrate the effects of surgery on immune cell subsets, including MDSC, Treg, CTL, and NK cells, in the lymphatic systems of patients with CRC, we analyzed variations in the MDSC, Treg, CTL, and NK cell populations in 26 patients with CRC before and after tumor resection. The proportions of CD33^+^CD11b^+^HLA-DR^−^ MDSCs and CD4^+^CD25^+^Foxp3^+^ Tregs among the PBMCs were significantly reduced in the CRC patients after the operation (*P* = 0.0001 and *P* = 0.0012, respectively), whereas no significant differences in the proportions of CD3^+^CD8^+^ CTL or CD3^−^CD16^+^ NK cells among the PBMCs were found before versus after tumor resection (Figure 3).

#### CRC cells promote the induction of MDSC *in vitro*

**Figure 4:**
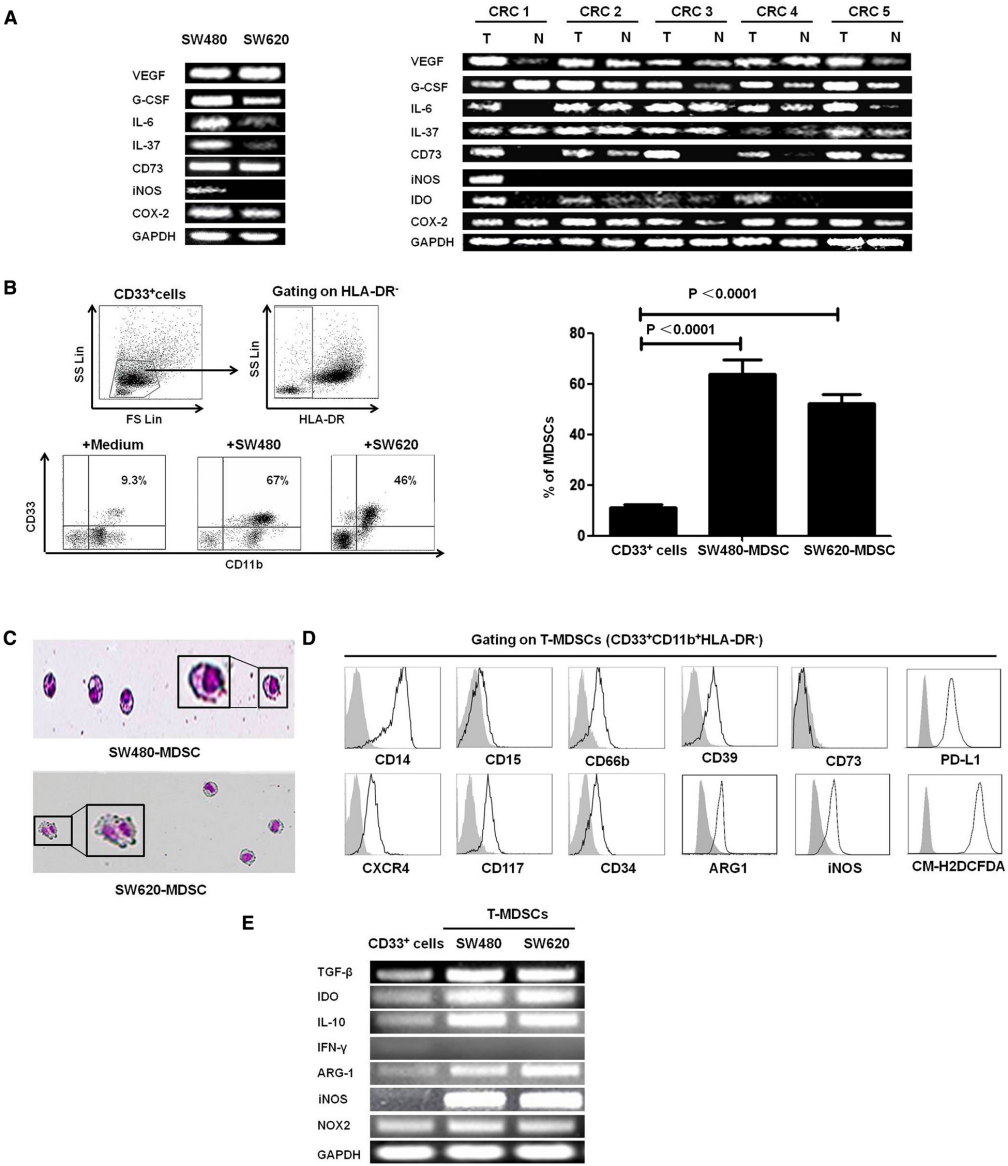
**Molecular characteristics of CRC cells and the induction of MDSCs via SW480 or SW620 cells** ***in vitro.*** **(A)** The expression of VEGF, G-CSF, IL-6, IL-37, CD73, iNOS, IDO, and COX2 in in SW480 and SW620 cells and in tumor and paraneoplastic tissues from 5 CRC patients was measured using quantitative RT-PCR. GAPDH expression was included as a control. **(B)** CD33^+^ cells were isolated from healthy PBMCs using human CD33 MicroBeads and were co-cultured with SW480 or SW620 cells. The representative dot plots and statistical graph show the proportion of HLA-DR^−^CD33^+^CD11b^+^ MDSCs induced from CD33^+^ cells by co-culture with SW480 or SW620 cells in a Transwell system for 48 hours. The CD33^+^ cells in medium alone were included as a control. **(C)** A representative cytospin of HLA-DR-CD33 + CD11b + MDSCs stained with Wright-Giemsa and identified by the mononuclear or polymorphonuclear cell nuclear staining (purple) using light microscopy (20 × 0.30 objective magnification) (Nikon Tokyo, Japan). **(D)** The phenotypes of the tumor-induced MDSCs were analyzed with flow cytometry using multiple anti-human mAbs against CD14, CD15, CD66b, CD39, CD73, CXCR4, CD117, CD34, Arg-1, iNOS, PD-L1 and ROS, and the grey curve represents autofluorescence as a negative control. Representative histograms are shown. **(E)** The mRNA levels of TGF-β, IDO, IL-10, IFN-γ, iNOS, Arg-1, and NOX2 in the T-MDSC by CRC cells, and CD33^+^ cells were detected using quantitative RT-PCR; GAPDH was included as control, and one of 5 experiments is shown here. T-MDSC, tumor-induced MDSCs by CRC cells.

Many studies have suggested that cytokines and other factors secreted by tumor cells can induce the generation of MDSCs. In agreement with this notion, high expression levels of VEGF, G-CSF, IL-6, CD73, iNOS, IDO, and COX-2 were observed in some tumor tissues and SW480 cells by semi-quantitative RT-PCR. In SW620 cells, the increase in the expression of genes was less significant, with only VEGF and COX-2 highly expressed. No expression of ARG-1 was detected in tumor or paraneoplastic tissues from patients or in SW480 or SW620 cells, and no expression of IDO was detected in SW480 or SW620 cells. Interestingly, iNOS expression was only found in tumor tissue from one patient (1/5) and in SW480 cells (Figure 4A).

Furthermore, we induced the generation of MDSCs from CD33^+^ PBMCs by culturing them with SW480 or SW620 cells in a Transwell System for 48 hours. The proportion of CD33^+^CD11b^+^HLA-DR^−^ MDSCs among the CD33^+^ PBMCs was significantly higher when CD33^+^ PBMCs were co-cultured with SW480 or SW620 cells in a Transwell System compared with CD33^+^ cells cultured in medium alone (Figure 4B). These CRC tumor-induced MDSCs were mononuclear or polymorphonuclear cells, as shown in Figure 4C by Wright-Giemsa staining. The CRC tumor-induced MDSCs exhibited high levels of CD14, CD66b, CD39, CXCR4, CD117, CD34, ARG1, INOS, ROS and PD-L1 expression but lacked CD15 and CD73 expression, similar to the MDSC phenotype observed in CRC patients (Figure 4D) Thus, CRC cells may promote the expansion of MSDCs *in vivo*. In addition, the CRC tumor-induced MDSCs expressed higher levels of immune inhibitory molecules, including TGF-β, IDO, IL-10, iNOS, NOX2, and Arg-1, and no IFN-γ expression compared with the CD33^+^ PBMCs cultured in medium alone (Figure 4E).

#### Tumor-induced MDSCs suppress the proliferation of T cells and promote the growth of CRC cell lines

**Figure 5:**
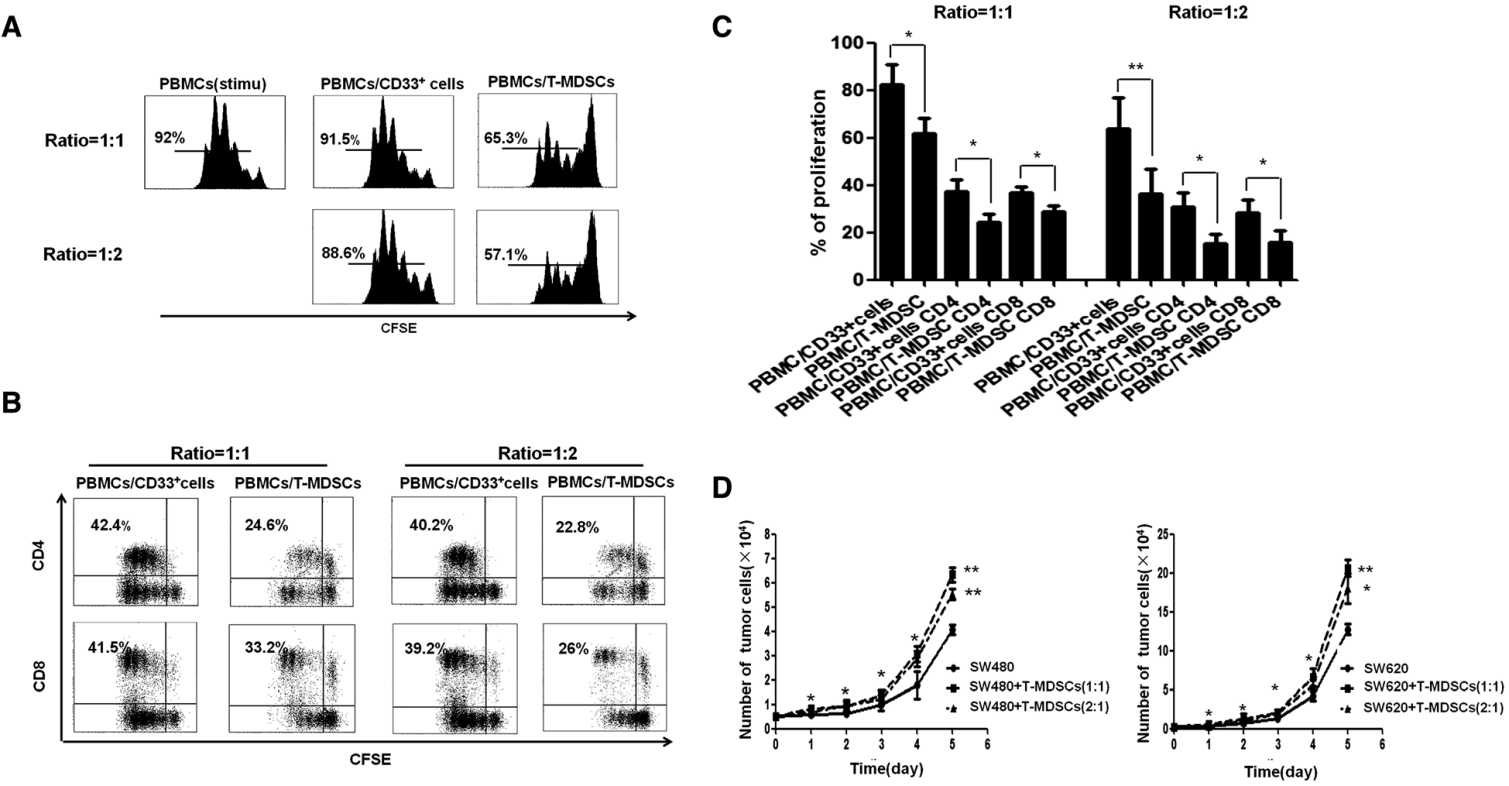
**The immunological and non-immunological functions of tumor-induced MDSCs.** The suppressive ability of the tumor-induced MDSCs on T cells *in vitro* was measured using a CFSE-labeled PBMC proliferation assay. M-MDSC: CD33^+^ cells cultured in medium alone; T-MDSC: tumor-induced MDSCs. The CFSE-labeled PBMCs were cocultured with M-MDSCs or T-MDSCs at a ratio of 1:1 or 1:2, respectively, in OKT3-coated 96-well plates. After 3 days, the cells were collected, stained with anti-human mAbs against CD4 and CD8 and quantified via flow cytometry. The proliferation of PBMCs, CD4^+^ T cells and CD8^+^ T cells was dramatically suppressed by T-MDSCs compared with M-MDSCs. (**A** and **B**) Shown are representative FACS density plots from one of 5 experiments, **(C)** A graph of the statistical analyses is shown. The error bars represent the SEM. *P < 0.05. **(D)** Tumor-induced MDSCs promoted the growth of SW480 and SW620 cells. SW480 or SW620 cells were seeded into a 96-well plate at 5 × 10^3^ or 2.5 × 10^3^ cells/well and cultured with T-MDSCs for 5 days at different ratios or in medium alone. The number of cells was counted every day. The statistical graph shows the mean value of three experiments. The number of SW480 or SW620 cells after coculture with T-MDSCs was significantly increased compared with the number of SW480 or SW620 cells cultured in medium alone. The error bars represent the SEM. *P < 0.05; **P < 0.01.

To investigate the function of these CRC tumor-induced MDSCs, we first analyzed the immunosuppressive function of the MDSCs on T cell proliferation in a co-culture system. In SW480 or SW620 cells, the tumor-induced MDSCs suppressed the proliferation of PBMCs more strongly than CD33^+^ cells cultured in medium alone; this suppression affected both CD4^+^ and CD8^+^ T cells (Figure 5A-C). In addition to their immunosuppressive function, these tumor-induced MDSCs promoted the growth of SW480 and SW620 cells in a coculture system *in vitro*, as shown in Figure 5D.

#### The function of tumor-induced MDSCs is mainly dependent on cell-to-cell contact and oxidative metabolism

**Figure 6:**
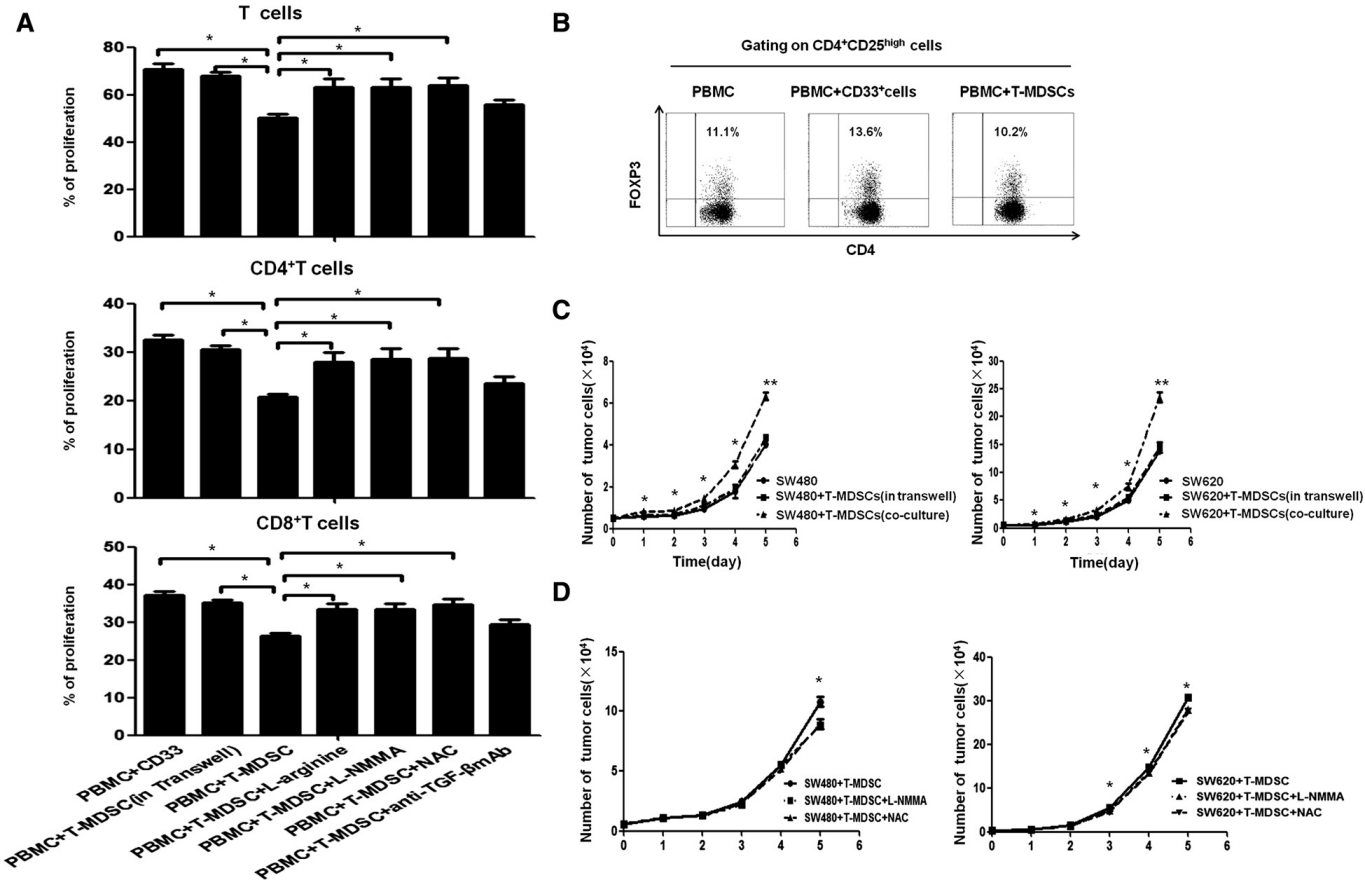
**The mechanisms of the immunological and non-immunological functions of tumor-induced MDSCs. (A)** The inhibition of T cell proliferation by tumor-induced MDSCs was rescued by a Transwell insert (put MDSC in inner well and PBMC in outer well) and inhibitors of L-Arg, iNOS and ROS, but not by anti-TGF-β. The statistical graph shows the mean value of 5 experiments. *P < 0.05. **(B)** CD4^+^ T cells were co-cultured with T-MDSC and CD33+ cells or in IL-2 medium alone in an OKT-3 stimulated 48-well cell plate for 3 days and then stained with anti-CD4, anti-CD25, and anti-FOXP3. The cells were analyzed using flow cytometry. One representative dot plot of three experiments is shown. **(C)** The tumor-induced MDSCs promoted the growth of SW480 and SW620 cells via a cell-to-cell contact-dependent mechanism. The cells were seeded into a 96 or 48-well plate at 5 × 10^3^cells/well and were co-cultured with T-MDSCs at a 1:1 ratio or were co-cultured with T-MDSCs in a Transwell system for 5 days. The number of SW480 or SW620 cells was significantly increased after co-culture with T-MDSCs compared with the number of SW480 or SW620 cells cultured in medium alone or with the number of SW480 or SW620 cells after co-culture with T-MDSCs in the Transwell system. **(D)** The promotion of CRC tumor-induced MDSCs on the growth of SW480 and SW620 cells was significantly decreased by the inhibitors of ROS and iNOS. The error bars represent the SEM. *P < 0.05; **P < 0.01.

We further investigated the molecular mechanisms of the function of these CRC tumor-induced MDSCs on T cells and tumor cells. For T cells, because the highly expressed inhibitory molecules on the MDSCs (Figure 4E) are linked to the suppression of T cell proliferation [31,32], we neutralized these inhibitory molecules by adding supplementary L-arginine, LNMMA, NAC and a neutralizing TGF-β antibody to the co-culture system. Supplementary L-arginine, LNMMA and NAC, which are inhibitors for iNOS and ROS, respectively, significantly reduced the immunosuppressive function of the CRC tumor-induced MDSCs (*P* < 0.05). However, neutralizing TGF-β antibody only slightly rescued the inhibition of CD4^+^ and CD8^+^ T cells by these tumor-induced MDSCs. In addition, the inhibition of CD4^+^ and CD8^+^ T cells by these tumor-induced MDSCs was reversed when a Transwell insert was used in the co-culture system of T cells and MDSCs, indicating that the immunosuppressive function of MDSCs was dependent on a cell-to-cell contact manner and oxidative metabolism which is in line with previous reports (Figure 6A) [14,33]. Moreover, the CRC tumor-induced MDSCs did not induce Treg cell expansion *in vitro* (Figure 6B). For tumor cells, we observed that the promotion of tumor growth induced by MDSCs was inhibited when the CRC cell lines SW480 and SW620 were co-cultured with tumor-induced MDSCs in a Transwell System (Figure 6C), indicating that the promotion of tumor cell growth by MDSCs is dependent on cell-to-cell contact. Next, we observed that the specific inhibitors LNMMA and NAC for iNOS and ROS, respectively, significantly reduced the promoting effect of CRC tumor-induced MDSCs on the growth of SW480 and SW620 cells (*P* < 0.05, Figure 6D).

### Discussion

The current studies examined the distribution and phenotypic markers of MDSCs in human CRC patients and how MDSCs interact with other cell populations in the CRC microenvironment, including cancer cells, cytotoxic T cells, and Treg cells. As with other human cancers, including breast cancer, gastric cancers, and murine cancers [34-36], we observed an expansion of MDSCs among PBMCs and tumorous tissues from patients with CRC. The increased circulating MDSC population correlated with advanced disease stages and tumor lymph node metastases in CRC patients. MDSCs typically express immature myeloid cell (iMC) markers, including CD34, CD33, the macrophage/DC marker CD11b, and IL4Rα (CD124); however, MDSCs typically do not express the lineage (Lin) markers of DCs or other mature myeloid cells [10,12]. Typically, the CD33^+^Lin^−^HLA-DR^-/low^ cells represent a general expanded MDSC subset in human cancers [37]. Here, our data suggest that the majority of the CD33^+^CD11b^+^ HLA-DR^−^ MDSCs observed in CRC consisted of a monocytic MDSC subset (CD14^+^CD15^−^CD33^+^CD11b^+^HLA-DR^−^) and an atypical granulocytic MDSC subset. These MDSCs express common human immature granulocyte-monocyte progenitor cell markers, including CD66b; CD117; CD34; the chemokine receptor CXCR4; CD39, an enzyme involved in the production of immunosuppressive adenosine; the immune mediators Arg-1, iNOS and ROS and the suppressive immune checkpoint molecule PD-L1; there was no expression of CD73. This finding contrasted with the phenotypic characteristics of MDSCs observed in other cancers, such as breast cancer [35], indicating that MDSCs are heterogeneous in different cancers. The analogous phenotypic characteristics of the MDSCs in peripheral blood and tumorous tissues from CRC patients and the high expression of CXCR4 on MDSCs indicated that the accumulation of MDSCs in tumorous tissues might be because of recruitment from the peripheral blood to tumors and aggregation *in situ*. A recent study discovered that CXCR2-expressing MDSCs could be tracked from the peripheral blood to the colonic mucosa according to their CXCR2 ligands, and these MDSCs were found to promote colitis-associated tumorigenesis in a mouse model of colitis-associated cancer. Additionally, the CXCL12-CXCR4 pathway triggered the accumulation of MDSCs to ovarian cancer ascites [23,38].

Correlations between the tumor burden and immune cells have been explored in cancer patients [39,40]. In the current study, we observed that the percentages of circulating MDSCs and Tregs were significantly decreased in patients after undergoing resection, whereas the percentages of CTL and NK cells were unchanged, indicating that immunosuppression is weakened by decreasing the MDSC and Treg cell populations in the CRC patients after reducing the tumor burden.

The direct interaction between MDSCs and tumor cells has been studied extensively, including in CRC [41,42]. In accordance with previous studies [41,43], high levels of VEGF, G-CSF, IL-6, CD73, iNOS, and COX-2 were observed in the tumorous tissues from CRC patients and in SW480 cells. Furthermore, in an *in vitro* experimental system, the CRC cell lines SW480 and SW620 could induce CD33^+^CD11b^+^HLA-DR^−^ MDSCs from CD33^+^ PBMCs. These tumor-induced MDSCs express high levels of immune inhibitory molecules, including TGF-β, IDO, IL-10, Arg-1, iNOS and NOX2, and could strongly suppress the proliferation of OKT3-stimulated CD4^+^ and CD8^+^ T cells. These data indicate that the CRC cells induce functional MDSCs *in vitro*, which is in agreement with previous reports for other types of cancer cells [17,44]. These tumor-induced MDSCs suppressed the proliferation of T cells and promoted the growth of SW480 and SW620 cells in a co-culture system *in vitro*, indicating that the mutual interaction of MDSCs with tumor cells as well as the interaction of MDSCs with T cells contributed to tumor development and disease progression in CRC. The promotion of tumor cell growth by MDSCs was recently reported in multiple myeloma [25]. Our data demonstrated for the first time that the promotion of tumor cell growth by MDSCs is dependent on a cell-to-cell contact mechanism in a Transwell System *in vitro*. Using neutralizing molecules, our data suggested that CRC tumor-induced MDSCs inhibited T cell proliferation and promoted CRC cell growth through oxidative metabolism, including the generation of NO and ROS, but not through TGF-β signaling or inducible Treg cells. Overall, these observations indicated that MDSCs promoted tumor cell growth through a direct interaction with tumor cells and the suppression of T cell anti-tumor immunity.

## Conclusions

The present study for the first time identifies a functional dependence between MDSCs, T cells and tumor cells in CRC: tumor cells induce the expansion of MDSCs via multiple inflammatory factors, and then these tumor-derived MDSCs suppress T cell proliferation and promote tumor cell growth through oxidative metabolism. Understanding the interactions between tumor cells and MDSCs may aid in the development of novel therapeutic approaches for CRC patients.

## Additional file

Additional file 1: Table S1. Baseline characteristics of patients. **Table S2.** The qRT-PCR primers for testing mRNA expression of interested genes.

## Abbreviations

Abbreviations: Full title
CRC: Colorectal cancer
MDSCs: Myeloid-derived suppressor cells
NO: Nitric oxide
ROS: Reactive oxygen species
TILs: Tumor-infiltrating lymphocytes
CTLs: Cytotoxic T cells
NKs: Natural killer cells
Tregs: Regulatory T cells
M-MDSCs: Monocytic MDSCs
G-MDSCs: Granulocytic MDSCs
FACS: Fluorescence-activated cell sorting
CM-H2DCFDA: 5-(and-6)-chloromethyl-2,7-dichlorodihydrofluorescein diacetate, acetyl ester
CFSE: 5,6 carboxyfluorescein diacetate, succinimidyl ester
L-NMMA: L-NG-monomethylarginine
NAC: N-acetylcysteine
ENTPD1: Ectonucleoside triphosphate diphosphohydrolase 1
ARG-1: Arginase 1
iMC: Immature myeloid cell
