# Supplementary material for: Tumor-induced myeloid-derived suppressor cells promote tumor progression through oxidative metabolism in human colorectal cancer
Source: J Transl Med. 2015 Feb 1;13:47. doi: 10.1186/s12967-015-0410-7 (PMC4357065; doi:10.1186/s12967-015-0410-7)
Supplement: Additional file 1: Table S1. — Baseline characteristics of patients. Table S2. The qRT-PCR primers for testing mRNA expression of interested genes. [file 12967_2015_410_MOESM1_ESM.doc]

**Table S1. Baseline characteristics of patients**

| **characteristics** | **No. of colorectal cancer patients (%)** |
| --- | --- |
| **Age(years)** |  |
| ≤60 | 27(64.3%) |
| ＞60 | 15(35.7%) |
| **Gender** |  |
| Male | 20(47.6%) |
| Female | 22(52.4%) |
| **TNM stage** |  |
| I | 3 (7.1%) |
| II | 21(50%) |
| III | 12(28.6%) |
| IV | 6(14.3%) |
| **T stage** |  |
| T1 | 0 |
| T2 | 3(7.1%) |
| T3 | 28(66.7%) |
| T4 | 11(26.2%) |
| **Tumor size(cm)** |  |
| ≤5 | 25(59.5%) |
| >5 | 17(40.5%) |
| **Lymph nodes metastasis** |  |
| No | 26(61.9%) |
| Yes | 16(38.1%) |
| **Histologic grade** |  |
| Well | 0 |
| Moderate | 24(57.1%) |
| Poor | 18(42.9%) |
| Undifferentiated | 0 |
| **Venous invasion** |  |
| No | 38(90.5%) |
| Yes | 4(9.5%) |
| **Serum CEA level(ng/mL)** |  |
| ≤5 | 22(52.4%) |
| >5 | 20(47.6%) |

**Table S2.** **The qRT-PCR primers for testing mRNA expression of interested genes**

| **Gene name** | **Primer Sequences** | **Product Size** |
| --- | --- | --- |
| VEGF | F: 5’- CACACAGGATGGCTTGAAGA-3’ | 135 |
|  | R: 5’-AGGGCAGAATCATCACGAAG -3’ |  |
| GCSF | F: 5’- GCAATGGGCACTGGGATGA-3’ | 333 |
|  | R: 5’-CGTCGGTGATGTTCGGGAG-3’ |  |
| IL-6 | F: 5’- AACCTGAACCTTCCAAAGATGG-3’ | 159 |
|  | R: 5’-TCTGGCTTGTTCCTCACTACT-3’ |  |
| IL-37 | F: 5’- TCTGCGGAGAAAGGAAGT-3’ | 99 |
|  | R: 5’-GCTGAAGGGATGGATGAC-3’ |  |
| CD73 | F: 5’-CCAGTACCAGGGCACTATCTG-3’ | 136 |
|  | R: 5’- TGGCTCGATCAGTCCTTCCA-3’ |  |
| iNOS | F: 5’-TTCAGTATCACAACCTCAGCAAG-3’ | 202 |
|  | R: 5’-TGGACCTGCAAGTTAAAATCCC-3’ |  |
| IDO | F:5’-TCAGGCAGATGTTTAGCAATG-3’ | 122 |
|  | R:5’-GGCACACGCTATGGAAAACT-3’ |  |
| COX2 | F: 5’- GTTTTGACATGGGTGGGAAC-3’ | 134 |
|  | R: 5’-CCCTCAGACAGCAAAGCCTA-3’ |  |
| ARG1 | F: 5’-GCAAGGTGGCAGAAGTCAAG-3’ | 165 |
|  | R: 5’-TTGTGGTTGTCAGTGGAGTGT-3’ |  |
| TGF-β | F: 5’-GGCCAGATCCTGTCCAAGC-3’ | 201 |
|  | R: 5’-GTGGGTTTCCACCATTAGCAC-3’ |  |
| IL-10 | F: 5-TCAAGGCGCATGTGAACTCC-3’ | 176 |
|  | R: 5-GATGTCAAACTCACTCATGGCT-3’ |  |
| INF-γ | F: 5-CTCTTGGCTGTTACTGCCAGG-3’ | 219 |
| NOX2(NCF1) | R: CTCCACACTCTTTTGGATGCT-3’  F:5-CCTGCCTCAATAGGGAACATT-3’  R:5-TCGTACCCAGCCAGCACTAT-3’ | 136 |
| GAPDH | F: 5’-CTCCTCCTGTTCGACAGTCAGC-3’ | 113 |
|  | R: 5’-CCCAATACGACCAAATCCGTT-3’ |  |

Note, F, forward primer; R, reverse primer
